# Assessment of the Relationship Between the Risk for Orbital Blowout Fracture After Trauma and Ethmoidal Sinus Morphometry Using the 3D Slicer Application

**DOI:** 10.3390/medicina62020266

**Published:** 2026-01-27

**Authors:** Meltem Özdemir, Handan Soysal, Erdem Özkan, Selcen Yüksel, Rasime P. Kavak

**Affiliations:** 1 Department of Radiology, Ankara Etlik City Hospital, University of Health Sciences, 06170 Ankara, Turkey; drrpelindemir6@hotmail.com; 2 Department of Anatomy, Faculty of Dentistry, Yıldırım Beyazıt University, 06010 Ankara, Turkey; handan_soysal@hotmail.com; 3 Department of Radiology, Kastamonu Training and Research Hospital, 37150 Kastamonu, Turkey; erdemozkan5454@gmail.com; 4 Department of Biostatistics, Faculty of Medicine, Yıldırım Beyazıt University, 06010 Ankara, Turkey; selcenyuksel@aybu.edu.tr

**Keywords:** head and neck trauma, orbital blowout fracture, ethmoid sinus, computed tomography, 3D slicer, morphometric analysis

## Abstract

*Background and Objectives:* The purpose of this study was to investigate whether high ethmoid sinus volume (ESV) constitutes a risk factor for the formation of orbital blowout fractures (OBFs) after craniofacial trauma and whether it affects the fracture pattern. *Materials and Methods:* This retrospective case–control study included patients aged ≥15 years who presented with craniofacial trauma to the emergency department of a Turkish university hospital between 1 October 2022 and 1 September 2023. The predictor variable was the presence of OBF (yes/no). The primary outcome variable was mean ESV, measured on computed tomography using the fully automated 3D Slicer software. Statistical analyses were performed with a significance level set at *p* < 0.05. *Results:* The case group consisted of 108 (median age: 41.5 years; 76 males, 70.38%), and the control group consisted of 122 (median age: 38 years; 84 males, 68.85%) subjects. OBFs were more frequent in males (69%), most commonly detected in the orbital floor (68.2%), and were bilateral in two (1.8%) subjects. The mean ESV in the case group (3.91 ± 1.39 cm^3^) was significantly higher than that in the control group (2.82 ± 0.94 cm^3^) (*p* < 0.001). Unlike the cases with medial wall fractures and those with orbital floor fractures, there was no significant difference in mean ESV between the cases with medial wall and orbital floor fractures and the control group (*p* = 0.562). *Conclusions:* A large ethmoid sinus not only increases the risk of orbital blowout fracture but also has an impact on the fracture pattern. Based on the data obtained from our study, we demonstrated a significant association between ethmoid sinus volume and the incidence of orbital blowout fracture.

## 1. Introduction

Orbital blowout fracture (OBF) is a relatively common type of maxillofacial fracture resulting from blunt trauma to the orbit. Like all other elements of the facial skeleton, the resistance of the bony orbits to trauma is provided mainly by the maxillofacial buttresses. The facial skeleton components that form the walls of the paranasal sinuses participate directly or indirectly in this buttress system. Thus, the highly complex paranasal skeleton, which varies considerably in size and shape among individuals, plays a role in maintaining the integrity of the facial bones [1,2]. There are publications in the literature demonstrating the relationship between frontal sinus dimensions and the incidence of anterior table frontal sinus fractures, and between maxillary sinus volume and the incidence of zygomatic fractures [3,4,5]. However, to our knowledge, no study investigating the relationship between ethmoid sinus volume (ESV) and the incidence of any facial fracture has been published to date.

**Figure 1:**
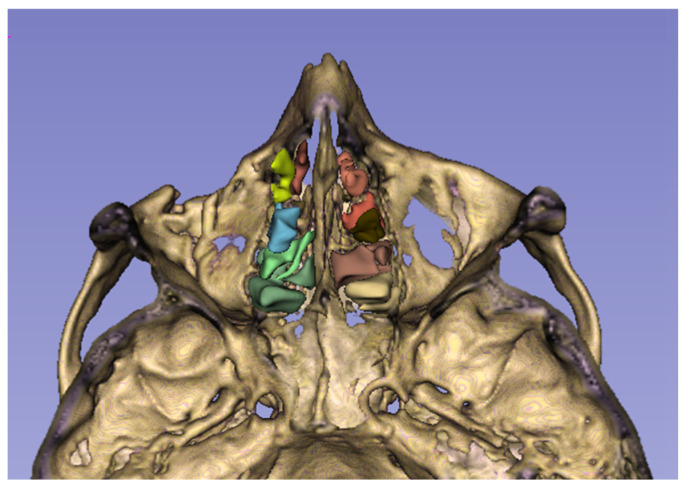
Axial view of the maxillofacial skeleton through the ethmoid sinus, with the ethmoid air cells colored. The figure was obtained by the 3D Slicer application.

The ethmoid sinus is a unique structure in that it contains multiple air chambers, unlike the other paranasal sinuses, which usually consist of a single air compartment. This special air space, located between the nasal and oral cavities, consists of multiple air cells formed by thin bones and separated from each other by the basal lamella of the middle turbinate [6] (Figure 1). Removal of ethmoidal septa during endoscopic sinus surgery has been shown to cause a change in the biomechanics of the orbit, leading to an increased risk of OBF, which is more pronounced in the medial wall than in the orbital floor [7,8]. We hypothesized that in a large ethmoid sinus, aligning the air cell walls relatively farther apart would reduce their buttressing effect for the orbital walls. The purpose of this study was to investigate whether increased ESV constitutes a risk factor for the occurrence of OBF following craniofacial trauma. Furthermore, we aimed to explore whether there is a relationship between ESV and OBF patterns.

## 2. Material and Methods

### 2.1. Study Sample and Design

Our study was initiated after receiving approval from the Ethics Approval Committee of the University of Health Sciences, Ankara Etlik City Hospital, Turkey (Approval date and number: 16 August 2023, 2023-484) and conducted in accordance with the Helsinki Declaration. Due to the retrospective design of the study, informed consent was not obtained. The radiological records of patients who were admitted to the Emergency Department following head trauma and underwent craniofacial computed tomography (CT) examination between 1 October 2022 and 1 September 2023 were retrospectively evaluated. The case group consisted of subjects with one or more OBFs in one or both orbits. The control group included subjects without any facial fractures. Since we aimed to make comparative analyses on paranasal sinuses that had reached their adult shape and size, the inclusion criterion for both groups was being over 15 years of age. The exclusion criteria for both groups were skull fracture and intracranial injury.

**Figure 2:**
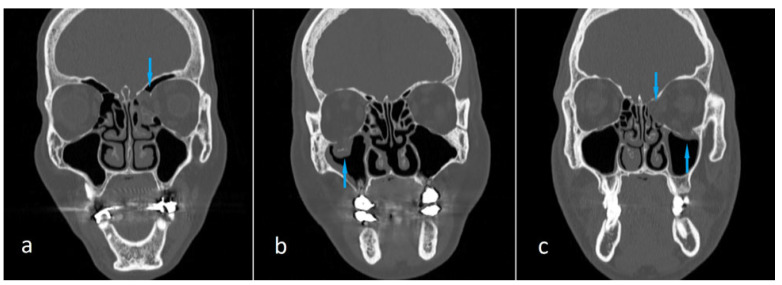
Coronal maxillofacial computed tomography images showing orbital blowout fractures located in the medial wall (**a**), orbital floor (**b**), and both the medial wall and orbital floor (**c**) (arrows).

The predictor variable was the presence of OBF (yes/no). The primary outcome variable was mean ESV, measured on computed tomography using the fully automated 3D Slicer software. Other study variables were classified into demographic (age, sex) and anatomic (fracture location) categories. We compared the case and control groups in terms of mean ESV. Sex was one of the two covariates of the study, and we compared case and control groups in terms of mean ESV separately for both women and men. The other covariate was the fracture location. The case group was divided into subgroups as follows: medial wall fractures, orbital floor fractures, and the fractures in both the medial wall and orbital floor (Figure 2). The mean ESV value of each subgroup was compared with that of the control group.

The “total bilateral volume” of the ethmoid sinus was calculated for each subject in the study population. Analyses performed in both the study group and subgroups were based on the number of subjects, regardless of the number of fractures detected in the patient, and calculations were made using the number of subjects as the basis.

### 2.2. CT Protocol and Volumetric Measurements with 3D Slicer

Imaging was performed by a 128-slice CT scanner (Revolution EVO, GE Healthcare System, Düsseldorf, Germany) using the following imaging parameters: 120 kV, 220 mAs, slice thickness = 0.625 mm, FOV = 18–24 cm. Reconstructions in three orthogonal planes with bone and soft tissue algorithms were used in the diagnosis of fractures. Fracture diagnosis and localization were determined separately by two radiologists experienced in the field of emergency radiology. In cases of conflicting diagnoses, the two radiologists evaluated the case together and reached a consensus.

We obtained the subjects’ CT images from our hospital’s Picture Archiving and Communication System (PACS) and saved them in Digital Imaging and Communications Medicine (DICOM) format. DICOM data were then transferred to a personal computer. 3D Slicer, an open-source software (https://www.slicer.org/, version 5.3.1), was used for analysis (accessed on 5 September 2025) [9]. The “total bilateral volume” of the ethmoid sinus was calculated for each subject in the study population.

**Figure 3:**
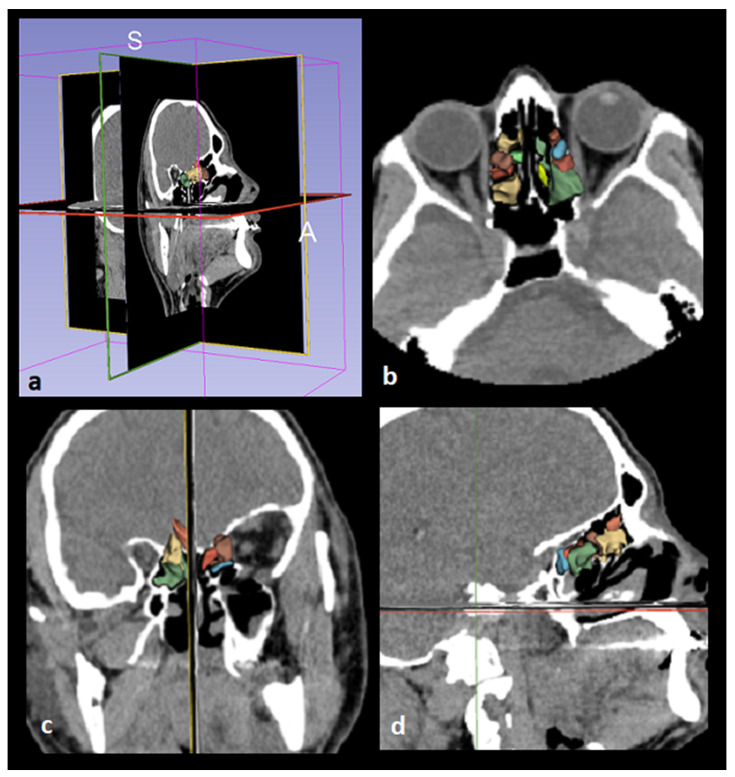
Images demonstrating the automated segmentation of the ethmoid sinus by the 3D Slicer application. Segmentation in three orthogonal planes is demonstrated in (**a**). Axial, coronal, and sagittal views are shown in (**b**), (**c**), and (**d**), respectively.

**Figure 4:**
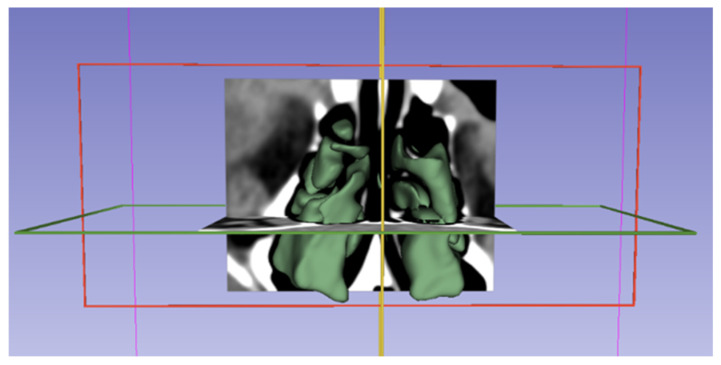
The 3D visualization of ethmoid sinus by the 3D Slicer data.

Images were displayed in the axial, coronal, and sagittal planes using the 3D Slicer application, and segmentation was performed (Figure 3 and Figure 4). The following steps were followed to select images suitable for segmentation and perform volumetric measurements.

1. The “Segment Editor” option was selected from the integrated tools in the “Modules” tab of the 3D Slicer screen.
2. New segmentation tabs were added with the “Add” toolbar.
3. In the first segment, the optimal spacing was set with the “Threshold” tool to encompass the anatomical boundaries of the ethmoid sinus (Threshold = −1500/−799 HU).
4. The raw images of the structures were displayed in three dimensions with the “Apply” command in the “View in 3D” tab.
5. The anatomical boundaries of the ethmoid sinus structures we wanted to study were examined, and any unsuitable areas were adjusted with the “Paint”, “Erase”, and “Scissors” tools.
6. A report showing the volume measurements of the structures was automatically obtained from the “Quantification” tab in the “Modules” table of the program.

All data segmentation was performed by two experienced anatomists using the same computer.

### 2.3. Statistical Analyses

Considering the effect size of 0.706 reported in medial wall measures and a Type I error rate of 0.05, the achieved statistical power of the study was calculated to be 0.99. SPSS version 20.0 was used to calculate the power of the study.

Age and ESV were compared between the case and control groups according to the distributional characteristics of the variables. Since the age variable did not meet the assumptions of normality, the Mann–Whitney U test was applied for group comparisons, and descriptive statistics were presented as median and interquartile range. For ESV, which showed a normal distribution, group comparisons were performed using the independent samples *t*-test, and descriptive statistics were expressed as the mean ± standard deviation. The chi-square test was used to assess the relationship between categorical variables. Frequencies and percentages were used to summarize categorical data. A Type I error level of 0.05 was adopted for all statistical tests. Statistical analyses were conducted using SPSS version 20.0.

Due to the insufficient number of medial wall and orbital floor observations in the female group, Monte Carlo Monte Carlo (MCMC) simulation-based results were reported. Frequencies and percentages were used to summarize the categorical data. Logistic regression analysis was conducted to identify predictors of the dependent outcomes. Separate binary logistic regression models were constructed for each dependent variable. The enter method was applied, and independent variables were entered simultaneously into each model. The Wald test was used to determine the statistical significance of individual coefficients. A Type I error level of 0.05 was adopted for all statistical tests. Statistical analyses were conducted using SPSS version 20.0 (IBM Corp., Released 2011, IBM SPSS Statistics for Windows, Version 20.0, Armonk, NY, USA).

## 3. Results

Baseline characteristics of the study sample are demonstrated in Table 1. The case group consisted of 108 subjects, and the control group consisted of 122 subjects. In the case group, there were 32 (29.62%) females and 76 (70.38%) males with a median age of 41.5 years (range: min 16, max 92). The control group consisted of 38 (31.15%) females and 84 (68.85%) males with a median age of 38 years (range: min 18, max 96). While both groups were predominantly male, there was no statistically significant difference between the two groups in terms of sex distribution (*p* = 0.803). No statistically significant difference between the case and control groups in terms of median age (*p* = 0.463) was noted.

In the examination of 108 subjects in the case group, two (1.8%) subjects had OBF in both orbits. Therefore, we identified a total of 110 fractures. Of the two subjects with bilateral OBFs, one had fractures in the medial walls, and the other had fractures in the medial walls and orbital floors. In the remaining 106 subjects, 36 (34%) had OBFs in the right orbit and 70 (66%) had in the left orbit. Of all the 110 fractures, 75 (68.2%) were located in the orbital floor, 18 (16.4%) in the medial wall, and 17 (15.4%) in both the medial wall and orbital floor. Multivariate analysis showed that there was no significant difference between females and males according to the fracture side, medial wall, orbital floor and medial wall, and orbital floor distribution (*p*: 0.698, 0.781, 0.403, and 0.347, respectively) (Table 2).

Comparison of the case and control groups in terms of mean ESV is shown in Table 3. The mean ESV in the case group (3.91 ± 1.39 cm^3^) was significantly higher than that in the control group (2.82 ± 0.94 cm^3^) (*p* < 0.001). Similarly, the mean ESV values in both males and females were significantly higher in the case group than in the control group (*p* < 0.001). Subgroup analyses showed that the mean ESV values of both the cases with medial wall fracture and those with orbital floor fracture were significantly higher than the mean ESV of the control group (*p*: 0.024 and 0.012, respectively). The association was more pronounced in the cases with orbital wall fractures compared to those with medial wall fractures. However, there was no statistically significant difference between the control group and the cases with medial wall and orbital floor fracture in terms of mean ESV (*p* = 0.562) (Table 4).

Summary of the logistic regression models is demonstrated in Table 5. We found that one-point increment in age increased the left side medial wall blowout fracture 1.046 times (*p* = 0.033; 95% CI: [1.004–1.090]). One-point increment in ESV increased the orbital blowout fracture 1.724 times (*p* < 0.001; 95% CI: [1.359–2.187]). By adjusting ESV, one-point increment in age increased the medial wall blowout fracture 1.026 times (*p* = 0.046; 95% CI: [1.001–1.052]).

## 4. Discussion

We retrospectively investigated the relationship between ESV and the incidence of OBF after craniofacial trauma. Making volumetric measurements of the ethmoid sinuses of subjects with and without OBF, we demonstrated that mean ESV was significantly higher in subjects with OBF compared with those without OBF (*p* < 0.001). This association was more pronounced in cases with orbital floor fracture (*p* = 0.012) compared to those with medial wall fracture (*p* = 0.024). However, no significant difference between the control group and the cases with medial wall and orbital floor fracture in terms of mean ESV was noted (*p* = 0.562). We found that OBF was more frequent in males (69%), and the most common fracture site was the orbital floor (68.2%). We detected bilateral OBFs in two (1.8%) subjects.

OBFs are isolated orbital wall fractures in which the orbital rim integrity is preserved. If not treated in time by the appropriate method, they can cause permanent functional and cosmetic disorders [10,11,12]. They occur as a result of a traumatic impact on the globe transmitted to the orbital walls. Assaults and falls have been reported as the two most common causes of OBFs [13,14]. They are most frequently located in the orbital floor, followed by the medial wall [15,16]. They occur more frequently in men than in women [16,17]. In accordance with the previous data, OBFs were more frequent in males (69%) and mostly located in the orbital floor (68.2%) in our study sample.

**Figure 5:**
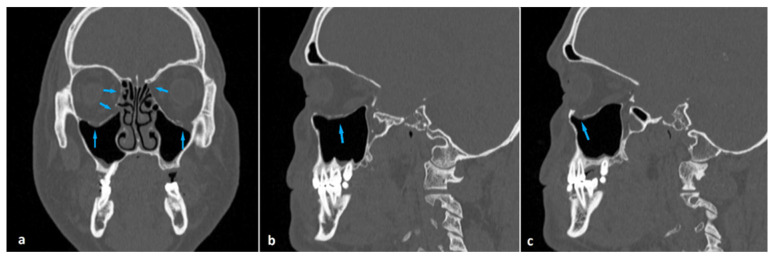
Coronal (**a**) and sagittal (**b**,**c**) maxillofacial computed tomography images of a 57-year-old man presenting with head trauma. He had bilateral blowout fractures (arrows) of the medial orbital walls and orbital floors (**a**). Right and left orbital floor fractures with herniation of fatty tissue into the maxillary sinuses (arrows) are shown in (**b**) and (**c**), respectively.

There are reports in the literature indicating that OBF, although rare, can occur bilaterally. Bilateral OBF has been reported to occur most commonly in older adults and as a result of exposure to high-energy trauma. The medial orbital wall has been shown to be more frequently affected in bilateral cases [15,18,19]. In our sample, there were two (1.8%) subjects with bilateral OBF. Both were male and above the median age of the case population. One had fractures in the medial walls, and the other had fractures in both the medial walls and the orbital floors (Figure 5).

Like other maxillofacial fractures, OBFs occur when a traumatic force beyond the resistance of the maxillofacial buttress system impinges on the face. The maxillofacial buttress system is mainly made up of four pairs of thick bony struts extending in vertical and horizontal planes and constitutes a unifying and protective structure that provides resistance of the facial skeleton against trauma. The medial orbital wall and the orbital floor are mainly supported by the medial vertical and upper transverse maxillary buttresses, respectively. Paranasal sinus walls align in continuity with this buttress system [10,11]. Previous data on ethmoid sinus biomechanics suggest that the uncinate process and ethmoid air cells serve as a buttress for the medial orbital wall, maintaining its integrity against trauma. This assumption explains why OBFs occur more frequently in the orbital floor, even though the medial orbital wall is thinner [7,8]. The ethmoid sinus typically consists of 7 smaller anterior and 4 larger posterior cells [6]. As ethmoid sinus volume increases, the surface area of the sinus and the distance between the ethmoid septae will increase. This configurational change can be expected to reduce the stabilizing effect of the ethmoidal septae on the orbital wall. Our results support the hypothesis that increasing ESV reduces orbital wall resistance and increases the risk of OBF.

In their retrospective study, Buller et al. focused on the stabilizing effect of the maxillary sinus on the zygomaticomaxillary complex. By comparing maxillary sinus dimensions in cases with and without zygomatic bone fractures, they found that the incidence of fracture was significantly higher in patients with greater sinus height and volume [5]. Two recently published studies on frontal fractures, which made similar assumptions about the relationship between paranasal sinus volume and fracture frequency, yielded similar results to the study on zygomatic fractures. They demonstrated a significant association between frontal sinus size and frontal fracture size and type [3,4]. The ethmoid sinus differs from other sinuses in terms of its central configuration and unique morphology. Because of its critical contact with the orbits, we addressed the effect of ESV on the resistance of the orbital wall to trauma in our study.

Unlike the cases with medial wall fractures and those with orbital floor fractures, we found no significant difference in mean ESV between cases with medial wall and orbital floor fractures and the control group. This finding implies that there is a difference between the mechanism of formation of the fracture pattern involving both the medial wall and orbital floor and the mechanism of the other two fracture patterns. This difference may be related to the cause of the trauma or the level of trauma energy transferred to the bone tissue. The momentum vector may also be another variable affecting the fracture configuration.

Over the past decade, we have witnessed the rapid development and widespread adoption of surgical methods used in the treatment of OBF. Studies contributing to the determination of the most appropriate surgical approach for each of the medial wall, orbital floor, and combined medial wall and orbital floor fractures, which were the subject of our study, are included in the literature. Transcaruncular, transconjunctival, and combined transconjunctival and transcaruncular surgical approaches are defined as current and safe surgical approaches in the treatment of OBF [20,21,22].

The main limitation of our study is that we did not evaluate the relationship between ESV and certain OBF characteristics, such as the degree of comminution, presence or absence of infraorbital canal injury, whether the fracture was accompanied by herniation, and presence or absence of rectus muscle injury. Additionally, we did not evaluate the potential impact of overall craniofacial size or ethmoid sinus anatomical variations on ESV. Another limitation of this study is that the mechanism of trauma, direction of impact, and the trauma energy were not addressed. Further comprehensive studies addressing the cause and the energy level of trauma are needed to draw accurate conclusions regarding the effect of ESV on the OBF pattern.

## 5. Conclusions

To our knowledge, this is the first study to investigate the relationship between ESV and the incidence of any facial fracture. The results of this study demonstrated a significant association between ESV and the incidence of OBF after craniofacial trauma. We found that a large ethmoid sinus not only increases the risk of OBF but also affects the fracture pattern. Based on the data obtained from our study, we demonstrated a significant association between ESV and the incidence of OBF.

## Figures and Tables

**Table 1:** Baseline characteristics of the study sample.

	Case Group	Control Group	*p* Value
**Sample size, *n***	108	122	
**Age, year (median, IQR)**	41.50 (27.75)	38.00 (33.00)	0.463
**Sex, *n* (%)**			
**Female**	32 (45.70)	38 (54.30)	0.803
**Male**	76 (47.50)	84 (52.50)	

*n*: Number, IQR: Interquartile range, *p*-values below 0.05 are considered significant.

**Table 2:** The distribution of blowout fracture side and location according to sex.

	Side and Location	Sex, *n* (%)		
		Female	Male	*p* Value
Side	Right orbit	10 (27.8)	26 (72.2)	0.698
	Left orbit	22 (31.4)	48 (68.6)	
Medial wall	Negative	64 (30.2)	148 (69.8)	0.781
	Positive	6 (33.3)	12 (66.7)	
Orbital floor	Negative	43 (27.7)	112 (72.3)	0.347
	Positive	27 (36.0)	48 (64)	
Medial wall and orbital floor	Negative	66 (31.9)	147 (68.1)	0.403
	Positive	4 (23.5)	13 (76.5)	

*n*: Number, *p*-values below 0.05 are considered significant.

**Table 3:** Comparison of the case and control groups in terms of mean ethmoid sinus volume.

Sex	Ethmoid Sinus Volume (Mean ± SD, cm^3^)		
	Case Group	Control Group	*p* Value
**Male**	3.88 ± 1.46	2.90 ± 0.98	<0.001 *
**Female**	3.96 ± 1.22	2.65 ± 0.84	<0.001 *
**Total**	3.91 ± 1.39	2.82 ± 0.94	<0.001 *

SD: Standard deviation, * Statistical significance.

**Table 4:** Comparison of the control group and case subgroups in terms of mean ethmoid sinus volume.

Fracture Site (*n*, %)	Ethmoid Sinus Volume (Mean ± SD, cm^3^)		
	Case Group	Control Group	*p* Value
**Medial wall (18, 16.4%)**	3.65 ± 1.37	2.82 ± 0.94	0.024 *
**Orbital floor (75, 68.2%)**	4.12 ± 1.81	2.82 ± 0.94	0.012 *
**Medial wall and orbital floor (17, 15.4%)**	2.21 ± 1.04	2.82 ± 0.94	0.562

*n*: Number, SD: Standard deviation, * Statistical significance.

**Table 5:** Summary of logistic regression models.

Dependent Variable	Predictor	B	*p*-Value	OR [Exp (B)]	95% CI (Lower–Upper)
Left side medial wall fracture	Age	0.045	0.033	1.046	1.004–1.090
Orbital blowout fracture	ESV	0.545	<0.001	1.724	1.359–2.187
Medial wall fracture	Age	0.026	0.046	1.026	1.001–1.052
	ESV	0.255	0.181	1.290	0.888–1.874

ESV: Ethmoid sinus volume, *p*-values below 0.05 are considered significant.

## Data Availability

The data presented in this study were derived from the following resource available in the public domain: [University of Health Sciences, Ankara Etlik City Hospital (URL: https://etliksehir.saglik.gov.tr) (accessed on 27 September 2025)].
